# Preparation and Properties of a Novel High-Toughness Solid Propellant Adhesive System Based on Glycidyl Azide Polymer–Energetic Thermoplastic Elastomer/Nitrocellulose/Butyl Nitrate Ethyl Nitramine

**DOI:** 10.3390/polym15183656

**Published:** 2023-09-05

**Authors:** Jing Zhang, Zhen Wang, Shixiong Sun, Yunjun Luo

**Affiliations:** 1 School of Materials Science and Technology, Beijing Institute of Technology, Beijing 100081, China; 2 School of Chemistry and Chemical Engineering, North University of China, Taiyuan 030051, China; 3 Dezhou Industrial Technology Research Institute of North University of China, Dezhou 253034, China

**Keywords:** GAP-ETPE/NC/Bu-NENA blend adhesive, Bu-NENA plasticizer, high toughness

## Abstract

Glycidyl azide polymer (GAP)–energetic thermoplastic elastomer (GAP-ETPE) propellants have high development prospects as green solid propellants, but the preparation of GAP-ETPEs with excellent performance is still a challenge. Improving the performance of the adhesive system in a propellant by introducing a plasticizer is an effective approach to increasing the energy and toughness of the propellant. Herein, a novel high-strength solid propellant adhesive system was proposed with GAP-ETPEs as the adhesive skeleton, butyl nitrate ethyl nitramine (Bu-NENA) as the energetic plasticizer, and nitrocellulose (NC) as the reinforcing agent. The effects of the structural factors on its properties were studied. The results showed that the binder system would give the propellant better mechanical and safety properties. The results can provide a reference for the structure design, forming process, and parameter selection of high-performance GAP-based green solid propellants.

## 1. Introduction

Double-base (DB) propellant, one of the earliest developed propellant varieties, is a homogeneous propellant with nitrocellulose as the adhesive and nitroglycerin as the plasticizer. It has been widely applied in solid rocket motors due to its various advantages, such as smokeless products, adjustable energy, abundant sources of raw materials, and mature technology [1]. However, DB propellants have the drawbacks of low-temperature embrittlement, largely caused by semirigid nitrocellulose (NC) macromolecules, and limited energy due to the limited concentration of oxidizer fragments [2,3]. Al and nitramines such as hexogen (RDX) [4], octogen (HMX) [3,5,6], and hexanitrohexaazaisowurtzitane (HNIW or CL-20) [2,5,7] are incorporated into propellant compositions to achieve higher performance. In this way, a modified double-base (MDB) propellant with increased specific impulse (I_sp_) was achieved [2], as expected. However, the presence of nitroglycerin (NG) and some high-energy materials in MDB propellants makes these systems extremely sensitive [2,5,8,9]. What is more, the toughness of the propellants worsens.

Because of their high production efficiency and low cost, DB and MDB propellants are important components of weapons and equipment. However, their poor safety performance introduces higher risks to their production, transportation, and use. The resulting safety accidents which occur from time to time are mainly caused by two factors: First, a large amount of NG is used as the energetic plasticizer in this type of propellant. It is extremely sensitive to mechanical stimuli and not conducive to the good safety performance of the propellant [10,11]. Second, nitrocellulose (NC), which is used as the adhesive skeleton in these propellants, has high molecular rigidity and strong intermolecular forces, resulting in a high processing temperature for the propellant, complicating its safe formation. The toughness is also very poor, especially for high-energy propellants with solid particles. Therefore, it is of vital importance to improve safety performance through the innovation of formulation components.

**Figure 1:**
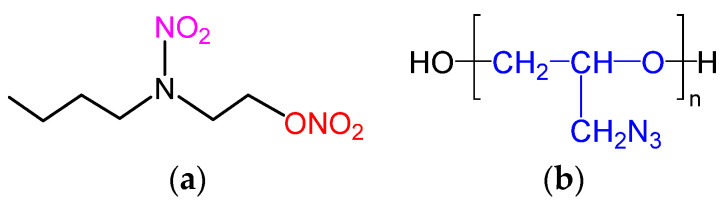
Structural formula of (**a**) Bu-NENA and (**b**) GAP molecule.

Several meaningful explorations have been carried out to reduce the mechanical sensitivity of DB and MDB propellants and improve the processing technology, including solid-filler coating, the optimization of the adhesive structure, and the introduction of additives [1]. Among them, optimizing the adhesive structure is one of the most effective ways to achieve these aims and is performed through the regulation of plasticizers and adhesives [12]. For plasticizers, butyl nitrate ethyl nitramine (Bu-NENA; the structure is shown in Figure 1), nitriethylene glycol (TEGDN), 1, 2, 4-butanol trinitrate (BTTN), trimethylethane trinitrate (TMETN), and other nitrate plasticizers mixed with NG or used alone can achieve the expected goal [1,2]. From molecular structural analysis, it can be determined that the same nitrate group that comprises Bu-NENA has an affinity with NC. Compared with NG, Bu-NENA, which has fewer nitrate groups and more flexible butyl, more easily enters the NC system to plasticize it. For sensitivity, as is known, the nitrate groups in NG are too close, and the groups easily influence each other, resulting in NG’s sensitive nature. Its characteristic drop height H_50_ is usually determined as less than 10 cm. For Bu-NENA, there is only one nitrate in a single molecule. The nitroamine group is much more stable than the nitrate group, and the molecule has a relatively long inert butyl group. Consequently, the mechanical sensitivity is significantly lower than that of NG, and the H_50_ can reach more than 110 cm. Therefore, Bu-NENA has a stronger plasticizing ability than NC and a lower mechanical sensitivity [13,14], which can improve the process performance of the system significantly [15,16,17]. For adhesives, the structural characteristics of NC can realize a narrow range of adjustable process performance. As a result, energetic polymer adhesives with better mechanical properties are available.

**Figure 2:**
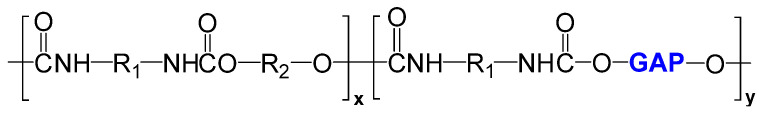
Structural formula of GAP-ETPE molecule.

Energetic thermoplastic elastomer (ETPE) is a two-phase block copolymer consisting of a soft segment and a hard segment, in which the soft segment provides softness and elasticity and the hard segment provides rigidity. These excellent properties make ETPE one of the best choices for an adhesive in propellants [18]. At present, the energetic polymers that have been developed are mainly nitrate polymers: azido-substituted oxysterol derivative polymers; azido cellulose and azide glycidyl ether polymers; and polymers containing nitroamines, nitro groups, and other energetic groups [15,16,17]. Among them, glycidyl azide polymer (GAP)–energetic thermoplastic elastomers (GAP-ETPEs) have good mechanical properties, low mechanical sensitivity, and a relatively mature preparation process. They are soluble and fusible, increasing their potential as green solid propellant binders. Therefore, developing new solid propellants using GAP-ETPEs as an adhesive remains a current research hotspot [17]. The structural formula of the GAP-ETPE molecule is shown in Figure 2, where R_1_ represents the carbon chain of the diisocyanate and R_2_ represents the carbon chain of the diol.

Based on the advantages of GAP-ETPEs and the urgent need for high-safety propellants in current weapon systems, this paper proposed a novel high-strength solid propellant adhesive system using GAP-ETPEs as the adhesive skeleton, Bu-NENA as the energetic plasticizer, and NC as the reinforcing agent. In our previous study [19], it was found that a GAP-ETPE/NC blend adhesive system experienced a sudden change in the performance of the blend system caused by the change in the NC state when the NC content was 30%. Therefore, in this paper, a GAP-ETPE/NC blend adhesive with 30% NC was selected as the base binder to prepare a GAP-ETPE/NC/Bu-NENA blend adhesive system, and the effects of the structural factors on its properties were studied. The results showed that the binder system was expected to improve the mechanical and safety properties of the propellant. These results can provide a reference for the structure design, forming process, and parameter selection of high-performance GAP-based green solid propellants.

## 2. Materials and Methods

### 2.1. Experimental Materials

The GAP-ETPE with 30% hard segment content synthesized in our laboratory has an average molecular weight of about 27,000. Its density was determined as 1.24 g/cm^3,^ and its enthalpy of formation was calculated as 4828 kJ/mol. The NC content was 12.0%; the average molecular weight was about 80,000 (Shanxi Xingan Chemical Plant (Taiyuan, China)). Bu-NENA, a light yellow oily liquid, had a melting point of −28.0 °C, density of 1.21 g/cm^3^, and heat of formation of 249 kJ/mol (Liming Chemical Institute, Luoyang, China).

### 2.2. Sample Preparation

**Figure 3:**
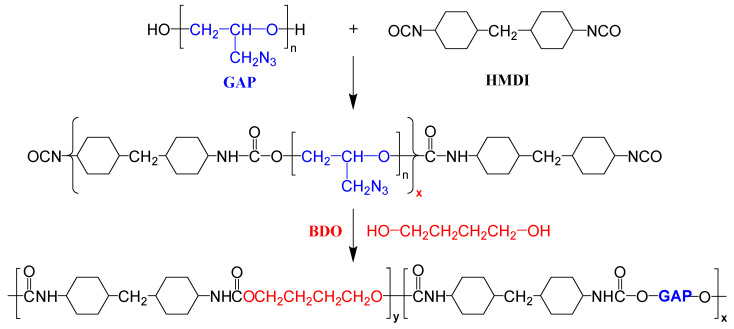
The synthesis process of GAP-ETPE with chains extended by BDO.

The synthesis strategy for GAP-ETPEs is shown in Figure 3. A detailed synthesis process can be found in Ref. [18]. Specifically, a stoichiometric amount of GAP was heated and stirred at 90 °C, and then a certain amount of catalyst and HMDI were added. The reaction mixture was stirred and mixed for 2 h at 90 °C. Then, BDO was added to the NCO-terminated GAP prepolymer after the system cooled to 60 °C. The resulting product was cast in a mold, kept for 3–5 min, and then cured at 100 °C for 12 h, after which the GAP-based ETPE with chains extended by BDO was finally obtained.

At 70 °C, the GAP-ETPE with a certain mass was calendered and plasticized for a certain time on a two-roll mixer, and then the Bu-NENA and NC were mixed, and the mixed Bu-NENA/NC was slowly added to GAP-ETPE in batches with repeated mixing, and then the mixture was calendered for 30 min after the GAP-ETPE was completely added.

The tablets obtained by rolling were placed in the mold, and the mold was placed in the middle position of the platen. At 70 °C, the mold was preheated for 30 min, and the equipment was started to close the mold at a molding pressure of 3–5 MPa for 2 min. The GAP-ETPE/NC/Bu-NENA samples were obtained by opening the mold. The sample with 10% Bu-NENA content was named E-N-N-10, and then E-N-N-15, E-N-N-20, E-N-N-25, and E-N-N-30 (Table 1), with the number indicating the Bu-NENA content. The sample without Bu-NENA was named N30, indicating that the mass percentage of NC was 30 wt%.

### 2.3. Experimental Instruments and Test Conditions

#### 2.3.1. Mechanical Property Test

The mechanical properties were tested with an AGS-J electronic universal testing machine (Shimadzu, Kyoto, Japan). Dumbbell splines were prepared according to GB/T528-1998 [20]. The test was performed at room temperature, and the tensile rate was 100 mm/min.

#### 2.3.2. Differential Scanning Calorimetry (DSC) 

A DSC1/500/578 differential scanning calorimeter (Mettler-Toledo, Zurich, Switzerland) was used over a temperature range of −100–100 °C at a heating rate of 10 °C/min. The sample mass was 4–5 mg. The sample was heated from room temperature to 100 °C, held for 10 min, then cooled to −100 °C, held for 10 min, and heated to 150 °C at a warming rate of 10 K/min. An N_2_ atmosphere was provided at a flow rate of 40 mL/min.

#### 2.3.3. Crosslinking Density Test

A magnetic resonance crosslinking density analyzer (Suzhou Niumai Electronic Technology Co., Ltd., Suzhou, China) was used to test the samples at different temperatures. Each sample was tested 5 times, and the average value was used.

#### 2.3.4. Scanning Electron Microscopy

An S4800 field-emission scanning electron microscope (Hitachi, Tokyo, Japan) was used for scanning electron microscopy (SEM) imaging with a cold field emission electron source, a resolution of 15 kV, and 500× magnification. The scale bar in the images is 100 μm.

#### 2.3.5. Rheological Test

The rheological measurements were performed on a modular advanced rheometer system (Haake, Vreden, Germany) equipped with a 20 mm parallel-plate geometry and a gap width of approximately 1 mm in air. The temperature was controlled with a Haake test chamber controller with an accuracy of ±1 °C. RheoWin Data Manager was used for equipment control, data acquisition, and analysis.

## 3. Results

### 3.1. Mechanical Properties of GAP-ETPE/NC/Bu-NENA Blend Adhesives with Different Bu-NENA Contents

The effect of Bu-NENA content on the mechanical properties of GAP-ETPE/NC/Bu-NENA adhesive systems was studied to improve the mechanical properties of the adhesives and expand the scope of mechanical property regulation of adhesive systems by providing a reference for the formulation design of high-performance propellants.

**Figure 4:**
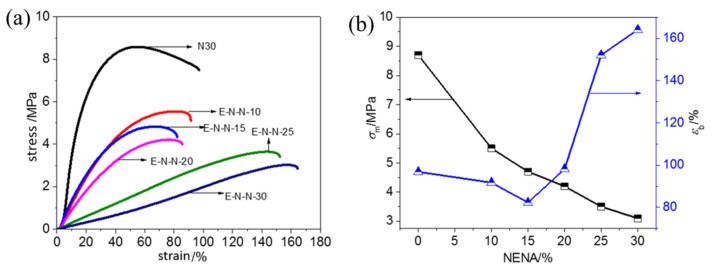
Tensile performance of GAP-ETPE/NC/Bu-NENA systems with different Bu-NENA contents. (**a**) Tensile curve; (**b**) maximum tensile strength and elongation at break. The test temperature was room temperature, and the tensile rate was 100 mm/min.

As shown in Figure 4 and Table 2, the maximum tensile strength observed for a GAP-ETPE/NC blend adhesive N30 was 8.7 MPa, which rapidly decreased with increased Bu-NENA. This is mainly because Bu-NENA not only destroyed the physical crosslinking network in the GAP-ETPE/NC system [21,22,23] but also increased the distance between the GAP-ETPE and the NC, reducing the strength of the intermolecular forces in the GAP-ETPE/NC system.

In addition, the elongation at break of the GAP-ETPE/NC/Bu-NENA blends decreased and then increased with increased Bu-NENA, mainly due to the plasticizing effect of Bu-NENA on NC and GAP-ETPE. For low Bu-NENA contents, the plasticization of NC and GAP-ETPE by Bu-NENA was incomplete, which reduced the tensile strength and elongation at break of the blend system. Moreover, a small amount of Bu-NENA could not completely plasticize the NC, and the size of the NC particles dispersed in the GAP-ETPE increased, resulting in stress concentration when the GAP-ETPE/NC/Bu-NENA blending system was subjected to external forces. Thus, the blending adhesive system was easily cracked and destroyed.

The experimental results show that in terms of mechanical properties, the room temperature strength of the adhesive system was adjustable in the range of 3.1–8.7 MPa, and the corresponding elongation was 96.8–164.1%. Generally, the tensile strength of the NC/NG adhesive system at 20 °C was about 10 MPa, and the elongation was about 20% (from DB propellant with NC/NG/C_2_ 51.5/47.5/1 mass%). Compared with the NC/NG adhesive system, the adhesive system proposed in this paper has a significant advantage in elongation, which is the performance factor critical to such propellants.

### 3.2. Thermal Transition Behavior of GAP-ETPE/NC/Bu-NENA Blend Adhesives with Different Bu-NENA Contents

**Figure 5:**
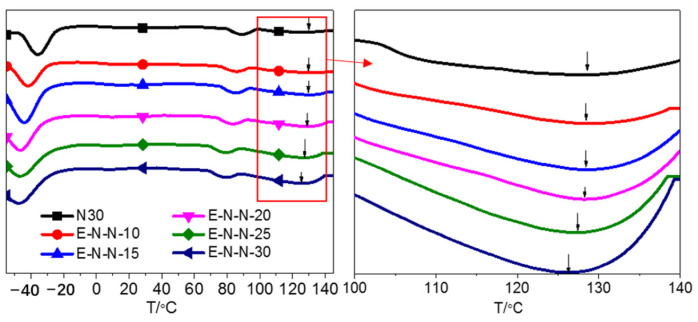
Differential curves of the DSC curves of GAP-ETPE/NC/Bu-NENA blend binders with different Bu-NENA contents. The heating rate is 10 k/min.

DSC was used to investigate the thermal transition behavior of GAP-ETPE/NC/Bu-NENA blend adhesives. Figure 5 and Table 3 show the differential curve and local magnification of the DSC curves and the glass transition temperatures of GAP-ETPE/NC/ Bu-NENA blend adhesives with different Bu-NENA contents, respectively. There were two transitions in the DSC curves of all the GAP-ETPE/NC/ Bu-NENA blend adhesives with different Bu-NENA contents before 120 °C, which was the glass transition temperature of the soft and hard segments in GAP-ETPE (Table 3). The T_g_^L^ of the GAP-ETPE/NC blend adhesives was −36.3 °C, and the T_g_^L^ of the GAP-ETPE/NC/Bu-NENA blend adhesives decreased with increased Bu-NENA content. When the Bu-NENA content reached 30%, the T_g_^L^ of the blend adhesives was −47.5 °C. 

The T_g_^H^ of the GAP-ETPE/NC/ Bu-NENA blends also decreased with increased Bu-NENA content due to the reduced intermolecular forces between the GAP-ETPE and NC from the addition of Bu-NENA [23]. This reduction made the molecular movement of GAP-ETPE and NC easier, reducing the T_g_ of the blend adhesive system. With increased Bu-NENA content, the plasticizing effect of Bu-NENA on GAP-ETPE and NC was enhanced, and the intermolecular forces between the GAP-ETPE and NC were further reduced, decreasing the glass transition temperature of the blend binder system.

Figure 5 shows a small peak (indicated by an arrow) appeared in the GAP-ETPE/NC/ Bu-NENA blend adhesive system above 120 °C. The peak became more prominent and moved to a lower temperature with increased Bu-NENA content. The peak may arise from the T_g_ of NC, between 170 °C and 180 °C [1]. In the blending system, Bu-NENA could dissolve and plasticize NC [23], and GAP-ETPE also has a plasticizing effect on NC [24], reducing the interaction forces between the NC molecules and between the NC and GAP-ETPE molecules. As a result, the molecular chain segments move easily, decreasing the T_g_ of NC.

Thus, Bu-NENA can not only plasticize NC but also effectively plasticize GAP-ETPE molecules, reduce the intermolecular forces in the system, increase the free volume of adhesive molecules, and enhance the motility of the adhesive system. This makes it a good candidate as a binder for solid propellants that will improve the toughness of the propellants.

### 3.3. Physical Crosslinking Density of GAP-ETPE/NC/Bu-NENA Blend Adhesives with Different Bu-NENA Contents

The crosslinking density of a polymer is closely related to the properties of the polymer system. The crosslinking density of the GAP-ETPE/NC/Bu-NENA system could reflect the number of physical crosslinking points in the system. The physical crosslinking points of this system include topological entanglement formed by mutual contact and entanglement among GAP-ETPE molecules, NC molecules, and Bu-NENA molecules, condensation entanglement formed by the interaction between local molecular chains (such as van der Waals forces and hydrogen bonding), and a small number of hard segment aggregation micro-regions in GAP-ETPE [23]. These points and the molecular chain segments of the blend system constitute the physical crosslinking network structure inside the blend adhesive.

**Figure 6:**
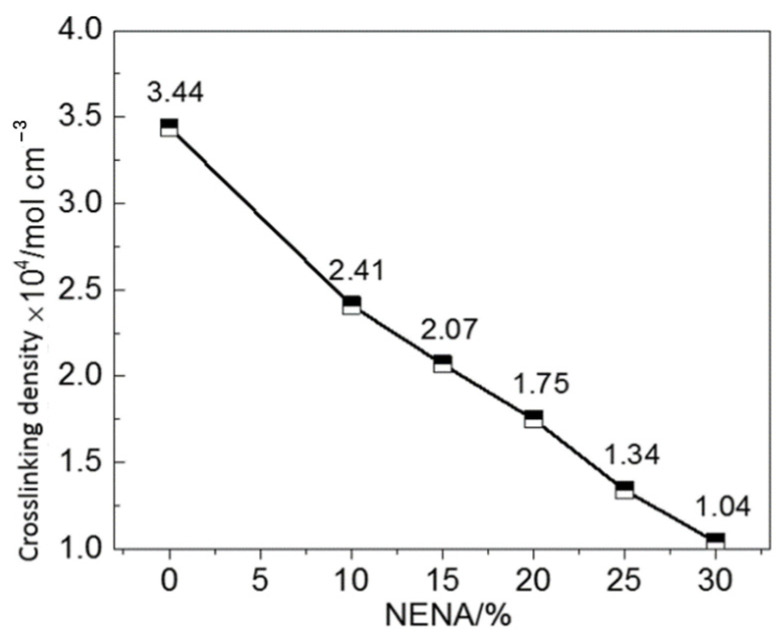
Crosslinking density of GAP-ETPE/NC/Bu-NENA blends with different Bu-NENA contents.

Figure 6 shows the crosslinking density of the GAP-ETPE/NC/Bu-NENA blends with different Bu-NENA contents. The physical crosslinking density of N30 (without added Bu-NENA) was 3.44 × 10^−4^ mol/cm^3^. With increased Bu-NENA content, the physical crosslinking density of the blend system continuously decreased. When the Bu-NENA content was 30%, the physical crosslinking density of the blend system decreased to 1.04 × 10^−4^ mol/cm^3^. The main reasons for this decrease were: (1) With the addition of the micro-molecule plasticizer Bu-NENA, the intermolecular force and friction between GAP-ETPE and NC were reduced, and the kinematic ability of the macromolecular chains of the blend system improved [22,23]. The hydrogen bonding and van der Waals forces formed between GAP-ETPE molecules, NC molecules, and GAP-ETPE-NC molecules in the system were destroyed, and the micro-region structure formed by GAP-ETPE hard segment agglomeration deteriorated, which reduced the number of condensed entanglement points in the system [25]. (2) Bu-NENA solvated the topological entanglement points formed between the GAP-ETPE molecules, NC molecules, and GAP-ETPE-NC molecules in the blending system, reducing the number of physical crosslinking points. (3) Bu-NENA increased the intermolecular distances of the blend system and hindered contact between molecular chains and the formation of physical crosslinking points, reducing the crosslinking density of the blend system [26]. In conclusion, the addition of Bu-NENA to the GAP-ETPE/NC system effectively reduced the number of physical crosslinking points and the role of the physical crosslinking network structure [27], affecting the viscosity, mechanical strength, and other properties of the blend system.

**Figure 7:**
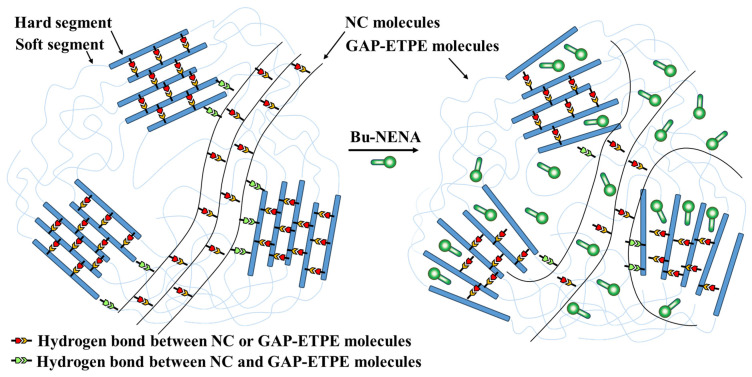
Schematic diagram of the influence of Bu-NENA on adhesive architecture.

The GAP-ETPE/NC/Bu-NENA adhesive system is complex, and the physical crosslinking points include topological entanglement points formed by mutual contact and intertwining of GAP-ETPE molecules, cohesive entanglement points formed by local molecular chain interactions (such as van der Waals forces and hydrogen bonding), and hard segment aggregation micro-regions with less content in GAP-ETPE. In order to represent the influence of Bu-NENA on the adhesive architecture, we use a schematic diagram and omit the ubiquitous van der Waals forces and entanglements that are difficult to represent simply. We mainly considered the influence of the hydrogen bonds and the hard segment aggregation regions, and the results are shown in Figure 7. In the absence of Bu-NENA, there were more hydrogen bonds between the hard segment aggregation regions of GAP-ETPE and the NC molecules, and there were also some hydrogen bonds between the GAP-ETPE and NC molecules. After the introduction of Bu-NENA, which can plasticize NC and GAP-ETPE, destroy the original hydrogen bonds, and increase the free volume between molecules, the motility of the GAP-ETPE and NC molecular chains improved. From the subsequent SEM imaging, when the content of Bu-NENA reaches a certain level, the NC diffused into the GAP-ETPE, improving the compatibility between the two and removing their phase interface.

### 3.4. Brittle Surface Morphology of GAP-ETPE/NC/Bu-NENA Blend Adhesives with Different Bu-NENA Contents

**Figure 8:**
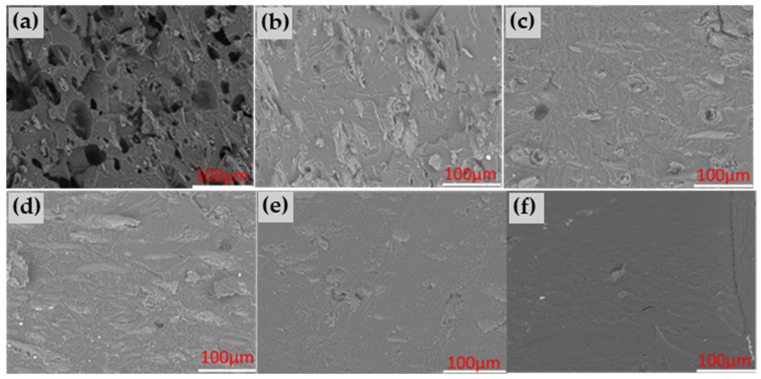
SEM photos of cross-sections of ETPE/NC/Bu-NENA blends with different Bu-NENA contents. (**a**) N30 does not contain Bu-NENA. (**b**) E-N-N-10, (**c**) E-N-N-15, (**d**) E-N-N-20, (**e**) E-N-N-25, and (**f**) E-N-N-30 are ETPE/NC/Bu-NENA blends. The samples were immersed in liquid nitrogen for 5 min and then broken manually, resulting in brittle fracture, before they were observed.

Figure 8 shows the SEM images of the brittle surfaces of GAP-ETPE/NC/Bu-NENA blends with different Bu-NENA contents.

As shown in Figure 8a, the surface of N30 is rough and uneven, with many holes and widely distributed particles. Moreover, the interface between the NC and the GAP-ETPE, caused by their poor compatibility, is obvious. Compared with the GAP-ETPE/NC blend adhesive, the number of holes on the surface of the GAP-ETPE/NC/Bu-NENA blend adhesive is significantly reduced, and the particle accumulation on the surface is larger, as shown in Figure 8b. This is mainly because the Bu-NENA was first mixed with the NC, which increased the plasticity and ductility of the NC. With increasing Bu-NENA content, some NC particles gradually entered the surface of the blend adhesive, and some particles protruded from the surface of the adhesive, resulting in visible fibrous NC, as shown in Figure 8c. With the further increase in Bu-NENA content, the number of particles on the surface of the GAP-ETPE/NC/Bu-NENA blend adhesive decreased, and the surface became flat with almost no raised particles, but an obvious fibrous profile was observed, as shown in Figure 8d. When the content of Bu-NENA increased further (Figure 8e,f), the particles on the surface of the GAP-ETPE/NC/Bu-NENA blend adhesive almost completely disappeared, and the surface became smooth and flat. The NC was completely plasticized by the high Bu-NENA content and changed from the solid state to the sol state, which came into more complete contact with the GAP-ETPE during the blending process. Moreover, Bu-NENA increased the free volume of the molecules in the blending system and the kinematic ability of the GAP-ETPE and NC molecular chains. The NC molecules were diffused into the GAP-ETPE molecules, resulting in better compatibility between the GAP-ETPE and the NC. The high concentration of Bu-NENA did not increase the tendency of the phases to separate. Instead, it improved the molecular interactions between the GAP-ETPE and the NC, enhancing their compatibility.

**Figure 9:**
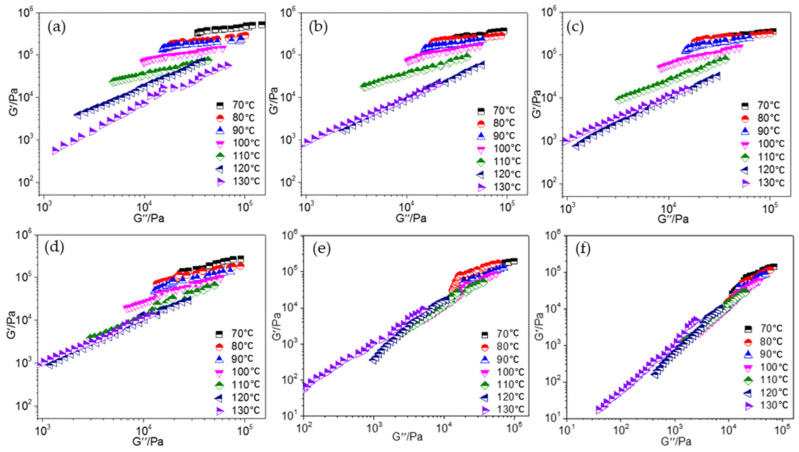
Han curve of the ETPE/NC/NENA-blended adhesive system: (**a**) N30, (**b**) E-N-N-10, (**c**) E-N-N-15, (**d**) E-N-N-20, (**e**) E-N-N-25, (**f**) E-N-N-30.

The Han curve (lgG″–lgG′ curve) [28,29] of the GAP-ETPE/NC/Bu-NENA blend adhesives was also studied to better understand the phase behavior. This curve is very sensitive to the morphological changes of multi-component or multiphase polymers and can be used to distinguish phase separations. Generally, the Han curves of homogeneous polymers at different temperatures can be superimposed to obtain a main curve, and there is no temperature dependence. For heterogeneous polymers (where phase separation exists in the polymer), the Han curves at different temperatures cannot overlap onto a main curve, which is temperature-dependent [28,29]. Studying the Han curve of a blended adhesive can help understand its phase change. Figure 9 shows the Han curve of the GAP-ETPE/NC/Bu-NENA-blended adhesive system at different temperatures. In Figure 9, the Han curves of the blending adhesive N30 are separated from each other at different temperatures and cannot be overlapped to form a single curve. There is an obvious temperature dependence, indicating a phase separation of GAP-ETPE and NC in N30. After adding Bu-NENA, the Han curves of the GAP-ETPE/NC/Bu-NENA system at different temperatures still do not overlap into a single curve, but the Han curves overlap at high temperatures. Above 120 °C, the Han curves of E-N-N-10 and E-N-N-15 overlap, and the Han curves of E-N-N-20 overlap at 110 °C when the Bu-NENA content is greater than 20%. The Han curves of Bu-NENA-blended adhesive systems almost overlapped at different temperatures. This is evidence that Bu-NENA compatibilized GAP-ETPE and NC in blending systems.

Combining the results of SEM imaging and Han curve analysis, Bu-NENA not only effectively plasticized NC and ETPE but also improved the compatibility between NC and GAP-ETPE. The phase separation in the adhesive was weakened, and the molecular mobility was enhanced. Finally, the toughness of the adhesive was significantly enhanced.

## 4. Conclusions

Based on the advantages of GAP-ETPE and the urgent need for high-safety propellants in current weapon systems, this paper proposed a novel high-strength solid propellant adhesive system using GAP-ETPE as the adhesive skeleton, Bu-NENA as the energetic plasticizer, and NC as the reinforcing agent. The crosslinking density, mechanical properties, and DSC showed that with increased Bu-NENA content, the crosslinking density and mechanical strength of GAP-ETPE/NC/Bu-NENA blends decreased monotonically, and the elongation at break first decreased and then increased. The T_g_ of the blending system decreased, and the T_g_ of NC was reduced to 120–140 °C. The results showed that this binder system is expected to give the propellant better mechanical and safety properties. These results provide a reference for the structure design, forming process, and parameter selection of a high-performance GAP-based green solid propellant.

## Figures and Tables

**Table 1:** GAP-ETPE/NC/Bu-NENA blend binders with different Bu-NENA contents.

Samples	GAP-ETPE/NC (7/3)/wt%	Bu-NENA/wt%
N30	100	0
E-N-N-10	90	10
E-N-N-15	85	15
E-N-N-20	80	20
E-N-N-25	75	25
E-N-N-30	70	30

**Table 2:** Mechanical property parameters of GAP-ETPE/NC/Bu-NENA systems.

Samples	*σ*_m_/MPa	*ε*_b_/%
N30	8.7	96.8
E-N-N-10	5.5	91.7
E-N-N-15	4.7	82.3
E-N-N-20	4.2	98.2
E-N-N-25	3.5	152.1
E-N-N-30	3.1	164.1

**Table 3:** T_g_ of GAP-ETPE/NC/Bu-NENA at N30 and different Bu-NENA contents.

Samples	T_g_^L^/°C	T_g_^H^/°C
N30	−36.3	89.4
E-N-N-10	−41.8	85.8
E-N-N-15	−44.5	85.1
E-N-N-20	−46.8	83.4
E-N-N-25	−47.0	80.3
E-N-N-30	−47.5	80.1

## Data Availability

Not applicable.
